# Prevalence and Associated Factors of Post-traumatic Stress Disorder in Gangjeong Village Residents, Jeju-do, Korea

**DOI:** 10.1192/j.eurpsy.2023.1847

**Published:** 2023-07-19

**Authors:** M.-D. Kim, Y.-E. Jung

**Affiliations:** Department of Psychaitry, Jeju National University Hospital, Jeju, Korea, Republic Of

## Abstract

**Introduction:**

Most studies on post-traumatic stress disorder (PTSD) have involved a small sample size and a specific traumatic event, with few studies reporting on subjects who have been continuously exposed to a traumatic event. Timely assessment and treatment are crucial for individuals chronically exposed to a traumatic event.

**Objectives:**

This study investigated the prevalence of PTSD and associated factors in all residents of Gangjeong village, who, recently, have been exposed to a traumatic event for a prolonged period.

**Methods:**

The subjects of this study were the residents of Gangjeong village, who have been exposed to a traumatic event related to the construction of the Jeju Civilian-Military Complex Port. The survey included items related to general characteristics and PTSD symptoms, which were assessed using the Impact of Event Scale-Revised, Korean version.

**Results:**

The prevalence of PTSD symptoms was 26.8% (95% confidence interval=23.54–30.04). Multivariate logistic regression analysis identified age, length of residence, and marital status as factors significantly associated with PTSD symptoms.

**Conclusions:**

The prevalence of PTSD symptoms was higher among the study population than in the general population. Economically active age groups, people exposed to the traumatic event throughout their duration of residence in the village, and unmarried individuals were found to be more likely to develop PTSD symptoms. Mental, social, and financial support should be directed to the affected groups of individuals.

**Disclosure of Interest:**

None Declared

